# Prevalence of treated patients with Alzheimer’s disease: current trends and COVID-19 impact

**DOI:** 10.1186/s13195-023-01271-0

**Published:** 2023-08-03

**Authors:** Javier Olazarán, Cristóbal Carnero-Pardo, Juan Fortea, Pascual Sánchez-Juan, Guillermo García-Ribas, Félix Viñuela, Pablo Martínez-Lage, Mercè Boada

**Affiliations:** 1 Unidad de Trastornos de La Memoria, HM Hospitales and Servicio de Neurología, HGU Gregorio Marañón, Madrid, Spain; 2 FIDYAN Neurocenter, Granada, Spain; 3 Memory Unit, Department of Neurology, Hospital de La Santa Creu I Sant Pau, Biomedical Research Institute Sant Pau, Universitat Autónoma de Barcelona, Barcelona, Spain; 4 Network Center for Biomedical Research in Neurodegenerative Diseases (CIBERNED), Instituto de Salud Carlos III, Madrid, Spain; 5 Fundación CIEN (Centro de Investigación de Enfermedades Neurológicas), Madrid, Spain; 6 Hospital Universitario Ramón y Cajal, Madrid, Spain; 7 Instituto Neurológico Andaluz, Hospital Victoria Eugenia, Seville y Unidad de Deterioro Cognitivo, Hospital Universitario Virgen Macarena, Sevilla, Spain; 8 Fundación Cita Alzheimer, Donostia-San Sebastian, Gipuzkoa, Spain; 9 Ace Alzheimer Center Barcelona, Universitat Internacional de Catalunya, Barcelona, Spain

**Keywords:** Alzheimer disease, Cholinesterase inhibitors, Incidence, Memantine, Spain, Prevalence, Treatment

## Abstract

**Background:**

There are few updated studies on the prevalence and management of Alzheimer’s disease (AD), which could be underdiagnosed or undertreated. The COVID-19 pandemic may have worsened the deficiencies in the diagnosis and treatment of these patients. Electronic medical records (EMR) offer an opportunity to assess the impact and management of medical processes and contingencies in the population.

**Objective:**

To estimate AD prevalence in Spain over a 6-year period, based on treated patients, according to usual clinical practice. Additionally, to describe the management of AD-treated patients and the evolution of that treatment during the 2020 COVID-19 pandemic.

**Methods:**

Retrospective study using the Spanish IQVIA EMR database. Patients treated with donepezil, galantamine, rivastigmine, and/or memantine were included in the study. Annual AD prevalence (2015–2020) was estimated and extrapolated to the national population level. Most frequent treatments and involved specialties were described. To assess the effect of COVID-19, the incidence of new AD cases in 2020 was calculated and compared with newly diagnosed cases in 2019.

**Results:**

Crude AD prevalence (2015–2020) was estimated at 760.5 per 100,000 inhabitants, and age-standardized prevalence (2020) was 664.6 (male 595.7, female 711.0). Monotherapy was the most frequent way to treat AD (86.2%), in comparison with dual therapy (13.8%); rivastigmine was the most prescribed treatment (37.3%), followed by memantine (36.4%) and donepezil (33.0%). Rivastigmine was also the most utilized medication in newly treated patients (46.7%), followed by donepezil (29.8%), although donepezil persistence was longer (22.5 vs. 20.6 months). Overall, donepezil 10 mg, rivastigmine 9.5 mg, and memantine 20 mg were the most prescribed presentations. The incidence rate of AD decreased from 148.1/100,000 (95% confidence interval [CI] 147.0–149.2) in 2019 to 118.4/100,000 (95% CI 117.5–119.4) in 2020.

**Conclusions:**

The obtained prevalence of AD-treated patients was consistent with previous face-to-face studies. In contrast with previous studies, rivastigmine, rather than donepezil, was the most frequent treatment. A decrease in the incidence of AD-treated patients was observed during 2020 in comparison with 2019, presumably due to the significant impact of the COVID-19 pandemic on both diagnosis and treatment. EMR databases emerge as valuable tools to monitor in real time the incidence and management of medical conditions in the population, as well as to assess the health impact of global contingencies and interventions.

**Supplementary Information:**

The online version contains supplementary material available at 10.1186/s13195-023-01271-0.

## Introduction

Dementia, mostly due to Alzheimer’s disease (AD), is one of the greatest global challenges for health and social care in the twenty-first century [1]. As AD evolves, the patient undergoes a progressive deterioration of cognitive abilities that leads to a gradual loss of autonomy, usually accompanied by significant affective and behavioral disturbances [2]. In fact, dementia is the main cause of institutionalization in the elderly [3] and is being increasingly reported as a predictor of death [4]. In developed countries, the number of older people with dementia is expected to rise more than double in the next 25 years [5].

Evidence indicates, and specialists agree, that underdiagnosis and undertreatment of patients with dementia are common, with serious consequences on patients’ functional capacity and quality of life [6–8]. To provide AD patients with adequate care, it is essential to proceed with timely detection, accurate diagnosis, and evidence-based management [9]. In that enterprise, professionals in health and social care fields should efficiently participate and interact with a common aim of maintaining patient functionality and quality of life, as well as reducing the caregivers’ burden [10].

With regard to the pharmacological treatment for AD, there are currently two types of marketed specific drugs: (a) cholinesterase inhibitors (ChEIs), i.e., donepezil, rivastigmine, and galantamine, and (b) a non-competitive *N*-methyl-d-aspartate (NMDA) receptor antagonist, i.e., memantine. For mild to moderate phases of AD, it is recommended that one of the three available ChEIs be used, whereas memantine is recommended, alone or in combination, in patients with moderate and severe AD [11]. These medications provide temporary symptomatic stabilization as well as a reduction in long-term mortality [12].

In Spain, as in many other countries, there is a deficit in the response to the needs of health and social services for the prevention, treatment, and care of Alzheimer’s dementia [13–16]. Due to these uncovered needs, a comprehensive plan was envisioned and published with the objective of promoting timely Alzheimer’s diagnosis and optimal management. Awareness programs were recommended for health professionals, focusing on the detection of signs and symptoms of neurodegenerative diseases and on the establishment of criteria-based, agile processes for patient referral to specialists from primary care [17].

The 2019 pandemic of coronavirus disease (COVID-19), caused by the severe acute respiratory syndrome coronavirus 2 (SARS-CoV-2), greatly stressed the healthcare systems worldwide. Since then, people with dementia have been consistently reported as the most vulnerable to the negative acute and post-acute consequences of SARS-CoV-2 infection [18, 19]. Among the significant changes that AD patients and healthcare professionals faced during the COVID-19 pandemic, many dementia consultations were withheld to either reduce transmission or redirect healthcare staff toward care for COVID-19 patients [20]. This likely led to the postponement of diagnosis and prescription in patients suffering from incipient dementia, thus increasing the anguish of the patient and the burden of the caregiver [21].

The primary objective of the study was to estimate the AD prevalence in Spain, in a 6-year period (i.e., 2015–2020), based on treated patients, according to usual clinical practice. Secondary objectives included (a) to describe the management of AD-treated patients (i.e., prescribed medications, average duration per treatment, and level of attended care specialization) and (b) to describe the evolution of AD treatment during the 2020 COVID-19 pandemic.

## Methods

### Study design

This was a retrospective, longitudinal, observational study. Patients’ data were extracted from the Spanish IQVIA Electronic Medical Records (EMR) database. This database contains the anonymized health records of patients from three Spanish regions, collected through 8000 office-based primary and secondary care physicians belonging to the public health system and covering approximately 3.0% of the Spanish population. The EMR population is comparable to the national population in age and sex (Additional file 1: Fig. S1).

For this study, AD was defined as the prescription of donepezil, galantamine, rivastigmine, and/or memantine, without a number of prescription limits. There were no age or sex restrictions for patient inclusion. Patient characteristics (age, gender), comorbidities (i.e., diagnostic code according to the International Classification of Diseases 9th Revision [ICD-9] and registration date), healthcare contacts (i.e., specialty and visit date), and prescriptions were collected. To qualify for a prescription, the medication had to be prescribed by the physician and dispensed in the pharmacy office. The analysis covered the period between January 1, 2013, and December 31, 2020, which was adapted according to database characteristics and specific study objectives.

The study protocol (version 0.1, May 11, 2021), with code ZAM-ALZ-2021–01, was approved by the *Hospital Clínic de Barcelona* Institutional Review Board (IRB), on May 21, 2021(meeting #10/2021).

### Statistical analysis

For the description of categorical variables, number, percentage of cases, and 95% confidence interval (CI) were calculated. For continuous variables, mean and standard deviation (SD) were obtained.

#### Prevalence

Annual AD prevalence was estimated by selecting the total number of Alzheimer’s patients that had at least one prescription of donepezil, galantamine, rivastigmine, and/or memantine during the year and extrapolating to the national population level, using Spanish population data provided by the National Institute for Statistics (NIS). Although data collection started in 2013, full harmonization between the three covered geographical regions was not achieved until 2015. For that reason, the period 2013–2020 was used for total patient detection, while the period 2015–2020 was selected to present annual and pooled prevalence.

It should be noted that the selection of treated Alzheimer’s patients was carried out on the base of patients treated annually, and from this total volume, those patients were defined as active. Hence, an active patient was prescribed annually with donepezil, galantamine, rivastigmine, and/or memantine. Then, the number of treated patients per year was extrapolated at the national level.

The period prevalence was estimated by dividing the mean number of cases obtained over the observation period (i.e., 2015–2020) by the population at the study’s midpoint on Donaldson’s epidemiology method [22]:

$$\mathrm{Period\, prevalence}=\frac{Mean\, number\, of\, active\, patients\, (extrapolated)}{Spanish\, population\, at\, the\, study's\, midpoint} \times \mathrm{100,000}$$

Age-standardized AD prevalence was obtained at the end of the study period (i.e., year 2020) using the European Standard Population 2013 (ESP 2013). In addition, the crude prevalence was obtained for the population aged 70 or more to propitiate comparison with previous investigations.

#### Incidence

The number of new AD-treated cases extrapolated from the database in 2019 and 2020 was utilized to estimate how the COVID-19 pandemic affected the diagnosis of AD.

$$Incidence=\frac{Number\, of\, new\, AD\, treated\, cases\, (extrapolated) }{Total\, Spanish\, population}\times \mathrm{100,000}$$

#### Comorbidities

The comorbidity analysis methodology was based on an extraction of all symptoms and diagnoses registered between 2013 and 2020 for patients previously identified as AD-prevalent, considering prevalent patients treated with donepezil, galantamine, rivastigmine, and/or memantine at any time during that period.

#### Patient management

With the purpose of describing the management of AD-treated patients (donepezil, galantamine, rivastigmine, and/or memantine), the following endpoints were considered:

- Number and percentage of patients that received treatment in 2020. This was calculated based on the monthly average. Monthly average was chosen as an adequate method to capture treatment diversity and modifications (i.e., treatment addition or abandonment, dose adjustment) during disease. Monthly treated patients were calculated based on the most representative treatment of each patient each month, which means the treatment received the highest number of days in that month. For a given treatment, the annual frequency was obtained by dividing the number of patients receiving that treatment as the most frequent (according to the annual average of the monthly days of treatment) by the number of analyzed (i.e., database active) patients.
  $$\mathrm{Annual\, frequency }=\frac{Number\, of\, patients\, receiving\, \left(most\, frequently\right)\, a\, given\, treatment}{Number\, of\, active\, patients}\times 100$$
- Number and percentage of patients who initiated donepezil, galantamine, rivastigmine, and/or memantine as treatment and maintained treatment or switched to a different medication. For this analysis, we selected those patients that initiated treatment between 2014 and 2017 and completed a follow-up period of 36 months. Treatment initiation was defined as the absence of (ChEI or memantine) prescription in the 12 months prior to the first prescription observed in the 2014–2017 period. Patients that switched treatment were defined as those who stopped using any treatment for AD and started any other treatments, including combinations. Additionally, the average duration per treatment (donepezil, galantamine, rivastigmine, and/or memantine) was analyzed and expressed in months (mean duration).
- Number and percentage of patients receiving the different presentations of the target medications (i.e., donepezil, galantamine, rivastigmine, and/or memantine) from January 2015 to December 2020. As treatments can be prescribed in monotherapy or as combination therapy, and a wide range of presentations are available in the current national market, the top ten, most used treatments were considered for the analysis. The mean number of treated patients’ monthly was obtained for every year, according to the selected treatment.
- Level of attended care specialization. The involved medical specialties were described for those patients that initiated treatment between 2014 and 2017 and completed a follow-up period of 36 months.

#### Impact of COVID-19

Newly diagnosed AD patients were defined as patients treated for the first time with any AD treatment (donepezil, galantamine, rivastigmine, and/or memantine), in 2019 and 2020.

## Results

### Study population

A total of 5001 prevalent AD patients were identified during the complete (i.e., 2013–2020) study period; of them, 64.1% were female and 35.9% were male. Age distribution was as follows: 0–59 years (0.8%), 60–69 years (4.6%), 70–79 years (28.4%), 80–89 years (55.8%), and ≥ 90 years (10.4%).

### Prevalence of AD

The prevalence of AD for the 2015–2020 period in Spain was estimated at 760.5 per 100,000 inhabitants (Table 1). Patients who received medications for treating AD were considered from the year 2015, as shown in Table 1. In general terms, the number of treated patients increased over recent years, particularly from 2015 to 2017. Overall, comparing 2015 and 2020, AD prevalence rose 1.48% (339,770 versus 344,805, respectively). However, from 2017 to 2020, the number of treated patients was somewhat lower (365,657 versus 344,805, respectively, 5.70% decrease).

**Table 1:** Prevalence of Alzheimer’s disease in Spain in 2015–2020

Year	Extrapolated treated patients (*n*)	Spanish population (*n*)
2015	339,770	46,410,149
2016	359,732	46,449,874
2017	365,657	46,532,869
2018	363,837	46,728,814
2019	354,098	47,105,358
2020	344,805	47,450,795
Mean	354,650	46,630,842^a^
2015–2020 prevalence per 100,000 population in Spain	760.5	

^a^At the study’s mean point

Crude and age-standardized prevalence is presented, for the year 2020, in Table 2. We observed an overall standardized prevalence of 664.6 (95% CI 662.4–666.8) of AD cases per 100,000 population in Spain. When age-standardized prevalence was estimated according to sex (Table 2), the prevalence in women was consistently higher than in men (711.0 per 100,000 women in the population [95% CI 708.1–714.0] versus 595.5 per 100,000 men in the population [95% CI 592.4–599.0]). In the sub-analysis of people aged 70 or more, the crude prevalence of AD was 4,801.6 (95% CI 4,773.5–4829.7), being the corresponding figures for women and men 5331.3 (95% CI 5001.2–5,361.4) and 4061.5 (95% CI 4035.3–4087.7), respectively. Age-specific AD prevalence and age-standardized cases, in the total population and stratified by sex, are listed in Additional file 1: Tables S1-S3.

**Table 2:** Crude and age-standardized AD prevalence (year 2020)

	**AD cases (*n*)**	**Crude prevalence per 100,000 population**	**Age-standardized prevalence per 100,000 population**	**95% CI for age-standardized prevalence**
Total population	344,805	726.7	664.6	662.4–666.8
Female	221,039	913.6	711.0	708.1–714.0
Male	123,766	532.2	595.7	592.4–599.0

*AD* Alzheimer’s disease, *CI* confidence interval

### Comorbidities

**Fig. 1:**
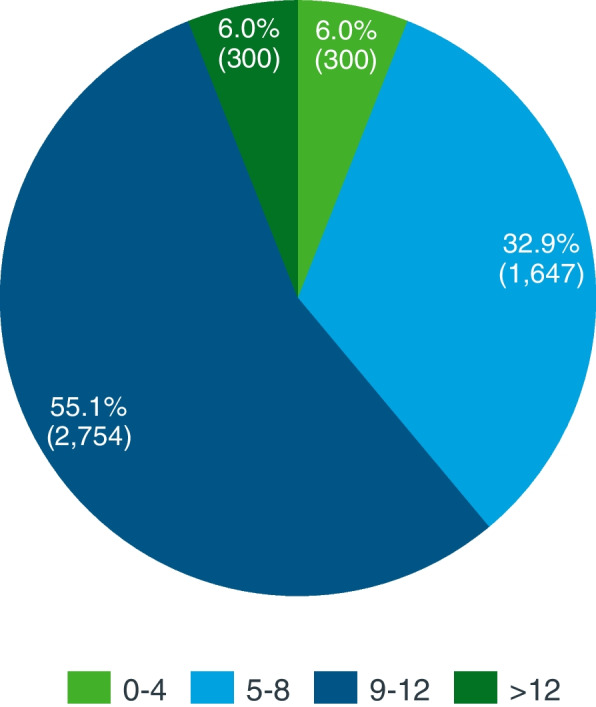
Percentage (and number) of Alzheimer patients with 0–4, 5–8, 9–12, and > 12 comorbidities (2013–2020 period, *n* = 5001 prevalent patients)

As expected, a wide range of comorbidities was associated with AD patients (Fig. 1). A percentage of 55.1% of patients were reported as having between 9 and 12 comorbidities, that being the most prevalent group of the categories; 32.9% of patients presented 5–8 comorbidities and the lowest percentage corresponded to the most extreme groups (6.0% of patients had 0–4 comorbidities and 6.0% of patients presented more than 12 comorbidities. The most prevalent comorbidity was sleep disturbance (codes 780–789, 91.6% of patients), followed by hypertensive disease (codes 401–405, 63.8%) and neurotic, personality, and other nonpsychotic mental disorders (codes 300–316, 63.2%) (see Additional file 1: Fig. S2 for a detailed description of the registered comorbidities).

### Management of patients with AD in Spain

#### Current treatment for AD

**Fig. 2:**
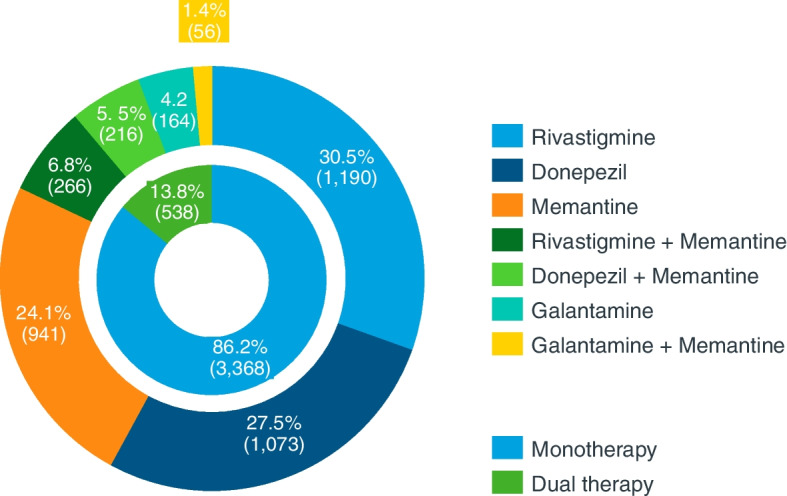
Distribution of Alzheimer patients according to therapy (year 2020, *n* = 3906 monthly treated patients)

According to our results, in 2020, rivastigmine was the most recurrent drug for treating AD in Spain, followed by donepezil and memantine. As Fig. 2 describes, monotherapy was the most frequent way to treat AD, at 3368 (86.2%), in comparison with combination therapy, at 538 (13.8%) patients. Considering patients on monotherapy, out of 3906 monthly treated patients, 1190 (30.5%) were treated with rivastigmine, 1073 (27.5%) received donepezil, and 941 (24.1%) patients were under memantine treatment. Galantamine was received by 164 (4.2%) of patients. The rest of the patients were receiving combination therapy, rivastigmine, and memantine being the most common combination among the patients, 266 (6.8%), followed by the combination of donepezil and memantine, 216 (5.4%) of patients. Galantamine and memantine combination in the treatment of Alzheimer’s corresponded to a rather low percentage of patients, 56 (1.4%).

**Fig. 3:**
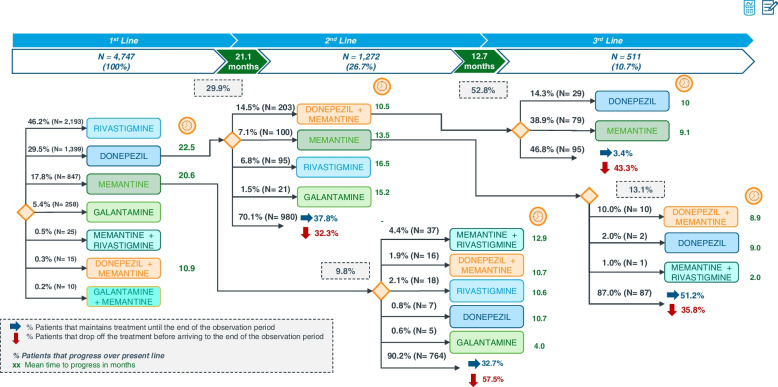
Treatment evolution in patients with donepezil or memantine as first therapy (follow-up period of 36 months)

**Fig. 4:**
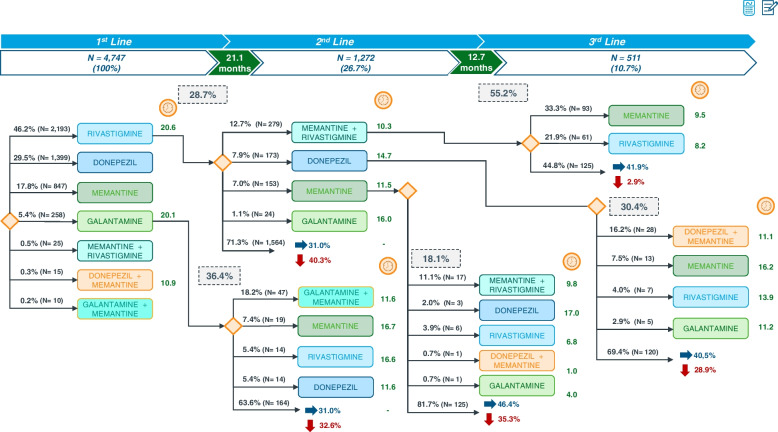
Treatment evolution in patients with rivastigmine or galantamine as first therapy (follow-up period of 36 months)

According to the EMR database, during a follow-up period of 36 months, a total of 4747 (100%) patients received a first-line treatment for a mean duration of 21.1 months. Of these patients, 1272 (26.7%) switched to an alternative therapy (second-line treatment), the mean duration of which was 12.7 months. Finally, a small percentage of patients, 511 (10.7%), who received a second-line treatment, switched to third-line therapy. The most frequent first-line treatment was rivastigmine (46.2%), followed by donepezil (29.5%), memantine (17.8%), and galantamine (5.4%). Treatment initiation with combination therapy (i.e., ChEI and memantine) was anecdotal (~ 1%). For a clear and detailed description of treatment evolution, the patients who received donepezil and memantine as first-line, as well as the subsequently prescribed treatment lines, are shown in Fig. 3, while the monitoring of patients who received rivastigmine and galantamine as first-line treatment is represented in Fig. 4.

For donepezil as the first line, the mean duration of treatment was 22.5 months. As shown in Fig. 3, 419 (29.9%) of those patients who received donepezil switched to another treatment alternative during the observation period: 203 (14.5%) received a combination of donepezil and memantine as second-line treatment for a mean duration of 10.5 months, and 100 (7.2%) received memantine as monotherapy, for 13.5 months. With regard to memantine treatment, 847 (17.8%) patients received it as the first line for a mean duration of 20.6 months. As expected, the number of patients that changed from memantine to a second-line treatment was low (9.8%) (Fig. 3).

Figure 4 describes the treatment flow of those patients who were administered rivastigmine, 2193 (46.2%), or galantamine, 258 (5.4%), as first-line treatment, as well as the progression to alternative treatments. The mean duration of rivastigmine treatment (20.6 months) was the same as that reported for memantine (Fig. 3) and close to galantamine (20.1 months). A total of 629 (28.7%) rivastigmine-treated patients switched to a second-line treatment, being a combination of memantine and rivastigmine the most frequent alternative therapy in this group of patients, 279 (12.7%), with a mean treatment duration of 10.3 months (Fig. 4). More than half of these patients, 154 (55.2%), switched to third-line therapy, memantine being the most prescribed drug, 93 (33.2%).

With regard to galantamine first-line treated patients, 94 (36.4%) of them switched to second-line treatment, a combination of galantamine and memantine being the most prescribed, at 47 (18.2%). However, the most durable second-line treatments for this group of patients were memantine (mean duration, 16.7 months) and rivastigmine (mean duration, 16.6 months), both as monotherapy (Fig. 4).

#### Treatment dosage

The most prevalent treatment prescriptions, as well as the mean number and percentage of patients that received the described prescription monthly, from 2015 to 2020, are presented in Table 3. If we observe the total number of patients who received a specific treatment, we can see an increasing tendency toward treatment prescriptions over the years, which ranges from a mean number of 3592 patients in 2015 to 3910 patients in 2020. However, the mentioned growth was not entirely linear, as in 2018 and 2020, small decrease in the mean number of total treated patients was observed (Table 3).

**Table 3:** Frequency of the different treatment presentations from 2015 to 2020^a^

Treatment and dose for the mean number of patients treated monthly	Year												Total mean (2015–2020)	
	2015		2016		2017		2018		2019		2020			
	*N*	%	*N*	%	*N*	%	*N*	%	*N*	%	*N*	%	*N*	%
Donezepil 10 mg	605	17%	689	18%	779	20%	819	21%	865	22%	855	22%	922	20%
Memantine 20 mg	661	18%	739	19%	802	20%	791	20%	748	19%	739	19%	896	19%
Rivastigmine Patch 9.5 mg	852	24%	826	21%	790	20%	710	18%	691	17%	705	18%	915	20%
Donezepil 5 mg	71	2%	100	3%	132	3%	180	5%	218	6%	218	6%	184	4%
Rivastigmine Patch 13.3 mg	90	2%	181	5%	196	5%	194	5%	190	5%	182	5%	207	4%
Rivastigmine Patch 4.6 mg	155	4%	159	4%	160	4%	173	4%	173	4%	180	5%	200	4%
Memantine 10 mg	53	1%	69	2%	86	2%	117	3%	165	4%	160	4%	130	3%
Donezepil 10 mg + Memantine 20 mg	128	4%	143	4%	128	3%	125	3%	133	3%	153	4%	162	3%
Memantine 20 mg + Rivastigmine Patch 9.5 mg	227	6%	171	4%	150	4%	132	3%	129	3%	120	3%	185	4%
Galantamine 24 mg	179	5%	168	4%	157	4%	135	3%	119	3%	109	3%	173	4%
Others	572	16%	607	16%	594	15%	544	14%	524	13%	491	13%	666	14%
**Total**	**3593**	**100%**	**3852**	**100%**	**3974**	**100%**	**3920**	**100%**	**3955**	**100%**	**3912**	**100%**	**4640**	**100%**

^a^Average number of monthly treated patients was obtained every year

According to our results, donepezil 10 mg, rivastigmine 9.5 mg, and memantine 20 mg would be largely the most prescribed treatment presentations in Spain, with global shares of 19–20% of total prescriptions. Focusing on the evolution of the described treatments from 2015 to 2020, we noticed that donepezil (10 mg and 5 mg), rivastigmine 13.3 mg, and memantine 10 mg were increased by 3–5% (absolute increase), and rivastigmine 9.5, galantamine 24 mg, and the combination of memantine 20 mg and rivastigmine 9.5 mg were decreased by 2–6% (absolute decrease), while memantine 20 mg, rivastigmine 4.6, and the combination of memantine 20 mg and donepezil 10 mg remained essentially unchanged (Table 3).

#### Patient flow chart according to specialty involved in diagnosis and monitoring of AD-treated patients

Neurology was the main specialty involved in the diagnosis of AD, with 3710 (78.2%) patients diagnosed and followed for a mean time of 14.3 months. A total of 1037 (21.8%) patients were diagnosed by other medical specialties, which included internists, geriatricians, psychiatrists, and general practitioners (GPs). Patients diagnosed by non-neurology medical specialties were monitored by that specialty during a mean time of 13.2 months.

**Fig. 5:**
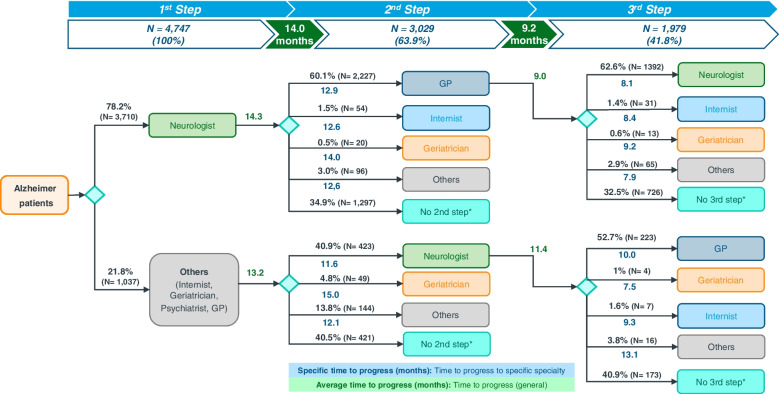
Patient journey according to specialties involved in diagnosis and monitoring of Alzheimer’s disease (follow-up period of 36 months). *Once the maximum time a patient can be followed up is reached, the patient flow terminates. GP, general practitioner

According to Fig. 5, follow-up of 60.1% (2227) of patients diagnosed by a neurologist was managed by GP, after a mean time of 12.9 months, while 34.9% (1297) of patients continued to be followed by neurologists. As for the patients diagnosed by non-neurology specialty, switch to neurologist was the most frequent change, which occurred in 40.9% (423) of patients, while 40.5% (421) did not change specialist. A second specialty switch (third step) was observed in more than half of patients, most frequently occurring between neurologist and GP: after a mean time of 8.1 months, 62.6% (1392) of patients that were followed by GP were switched to neurologist, while 52.7% (223) of patients that were followed by neurologist were switched to GP, after a mean time of 10.0 months (data of switches between other specialties are not shown).

### Impact of COVID-19 on Alzheimer’s diagnosis and treatment

The annual incidence of AD was estimated at 148.1 patients per 100,000 population (95% CI 147.0–149.2) in 2019, while the incidence estimation for the year 2020 was 118.4 patients per 100,000 population (95% CI 117.5–119.4). Hence, a significant decrease in the estimated incidence of AD, according to newly reported treated patients, was observed in 2020, coinciding with the COVID-19 pandemic (Table 4).

**Table 4:** Incidence of Alzheimer’s disease in Spain in 2019 and 2020

Year	New AD cases	Spanish population	AD incidence per 100,000 population in Spain	95% confidence intervals for incidence
2019	69,743	47,105,358	148.1	147.0–149.2
2020	56,086	47,351,567	118.4	117.5–119.4

**Fig. 6:**
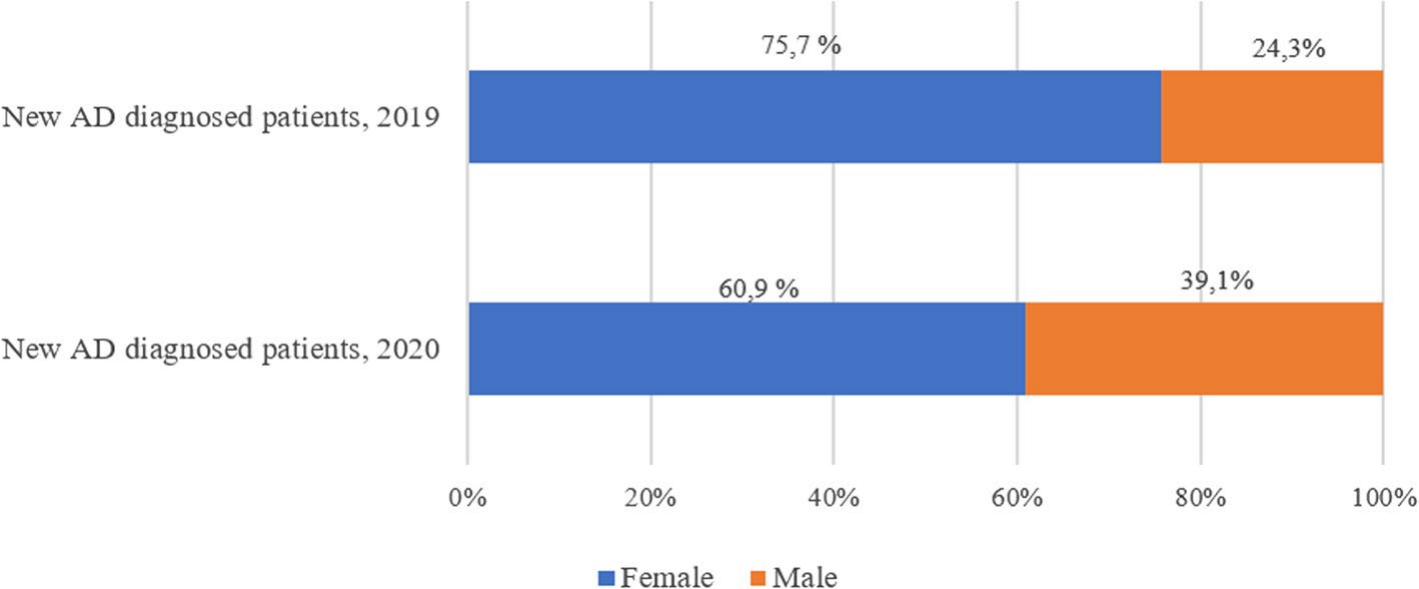
Newly diagnosed Alzheimer patients in 2019 and 2020 years, stratified by sex

Figure 6 shows the proportion of newly diagnosed AD patients by sex in 2019 and 2020 years. For both years, over half of new AD cases occurred among females. However, there was a decrease in the proportion of female patients diagnosed during 2020 (60.9%) in comparison with 2019 (75.7%), leading to an increase in the frequency of new male AD cases in 2020 (39.1%) versus 2019 (24.3%).

## Discussion

We interrogated a national EMR database to estimate the prevalence of patients treated for AD, to describe the pharmacological and specialty management, and to evaluate the impact of the COVID-19 pandemic on the provision of healthcare in Spain. According to the presented results, the age-standardized prevalence of AD during the 2015–2020 period was estimated at 760.5 per 100,000 inhabitants, which falls within the 700–799 prevalence interval obtained in the Global Burden of Diseases, Injuries, and Risk Factors (GBD) Study [23]. That similarity of results was unexpected, since non-AD dementias (vascular, Lewy body, frontotemporal dementias, etc.) were included in the GBD Study. Moreover, a recent study, also built on EMR data, found incidence and prevalence estimations of AD slightly lower than those obtained in face-to-face studies [24]. Nevertheless, a reanalysis of face-to-face dementia surveys, conducted in the population aged 70 and over, age- and sex-adjusted prevalence of AD ranged from 2.6 to 7.7% [25], which is consistent with our age-adjusted prevalence of 4.8%, obtained for the same group of age. We could speculate that, in our investigation, possible underestimation of AD prevalence due to the lack of diagnosis or treatment was counter-balanced by the prescription of ChEI and memantine to non-AD dementia patients, eventually providing a valid estimation of AD prevalence, incidence, and evolution. As in previous face-to-face studies [25], we obtained higher crude and age-standardized AD prevalence in women than in men, which gives further support to the utilized methodology.

The comorbidity of our “real-world” AD patients was remarkably high, with almost two out of three patients (61.1%) displaying nine or more associated medical conditions. That is not surprising since also two each of every three patients (66.2%) had 80 or more years of age. These results point to the need to investigate the potential medical and psychiatric modifiers of the clinical and biological course of AD, as well as how comorbidities can alter treatment response and quality of life, especially in older patients [26].

Rivastigmine, memantine, and donepezil, alone or in combination, were the most frequently used AD medications, which were prescribed to 94.4% of patients, while galantamine was only utilized in 5.6% of patients. Preponderance of donepezil over galantamine, rivastigmine, and memantine was reported in a retrospective study of EMR that analyzed GPs’ dementia patients and covered the period from 2005 to 2015 [27]. Similar results were obtained in a recent multinational study based on record form completion of AD patients, conducted by GPs, neurologists, geriatricians, and psychiatrists [28]. In contrast, we found a similar frequency of prescription of donepezil, rivastigmine, and memantine, which could be due to more advanced dementia stage or sample contamination with non-AD patients. Regarding ChEIs, reasons to favor the use of donepezil and rivastigmine, versus galantamine, may be related to ease of use and specific clinical effects, such as improvement of psychotic symptoms [29].

Overall, monotherapy prevailed over dual therapy (86.2% vs. 13.8%), which is consistent with previous investigations, although we obtained a slightly higher prevalence of combined treatment (13.8% vs. 6.0%), mostly due to the appearance of rivastigmine and memantine combination [28]. These low figures of dual therapy suggest suboptimal treatment, considering the good tolerance, prolonged clinical benefits, and institutional and expert recommendations favoring the use of ChEI and memantine combination [11, 30, 31].

With regard to the first-line treatment that AD patients received in Spain, ChEIs were the choice in most patients (81.1%), again consistent with the existing studies and guidelines, reporting very mild or unclear effects of memantine in mild AD [32]. However, in contrast with previous studies, rivastigmine was the most prescribed monotherapy (46.2%), followed by donepezil (29.5%), memantine (17.8%), and galantamine (5.4%). In a study of Medicare beneficiaries that initiated AD treatment during the period of 2001–2003, donepezil was the most frequent drug (62.8%), followed by galantamine (17.2%) and rivastigmine (20.1%) [33]. In a Canadian population-based study conducted during the period of 2009–2013, donepezil was also the most frequent medication (59%), followed by rivastigmine (22%) and galantamine (19%) [34]. A Korean study analyzing data of the 2009–2019 period obtained a prevalence of 48.8% (donepezil), 18.1% (memantine), 9.0% (rivastigmine), and 5.7% (galantamine) [35]. The higher frequency of use of rivastigmine observed in our investigation could be due to the inclusion of non-AD patients, particularly patients with Lewy body or Parkinson’s disease dementia [36] but could also indicate a new trend of prescription in real-world patients—where dementia etiology is not always clear—that favors the use of medications that offer a wide range of target symptoms, doses, and presentations [37, 38].

Despite the higher frequency of rivastigmine prescription, donepezil showed slightly better persistence: 37.8% of patients that started on donepezil maintained the treatment (versus 31.0% of patients that initiated treatment with rivastigmine), and the mean time from treatment initiation to switch was 22.5 months for donepezil (versus 20.6 months for rivastigmine). In previous studies of new ChEI users, rivastigmine was also associated with shorter mean persistence and higher switch rate, compared to donepezil [34, 35]. These results should be taken cautiously though, since the patients’ demographic and clinical characteristics (age, comorbidities, neuropsychiatric symptoms, etc.) were not controlled.

Treatment initiation by neurologists, along with patient transfer between GP and neurologist, was the most common specialty scenario, albeit a relevant number of patients were managed by other specialties. That is consistent with previous studies conducted in Spain [39] but not in other countries, where management by GP prevailed and the participation of other specialties was less frequent [40]. Clearly, in a context of varied region and country-specific scenarios, EMR studies may help to inform administrations and providers to achieve the so-needed global and coordinated care for people with dementia and their families [11, 17, 41].

The COVID-19 pandemic changed our lives profoundly in a very short time, provoking devastating effects on people with dementia [21]. As the number of professionals was reduced and most face-to-face consultations were canceled, medical efforts were focused on the symptomatic relief of the affective and behavioral symptoms of the already diagnosed patients, as well as on the advice of their relatives, usually through teleconsultations [19]. Not surprisingly, the incidence of AD-treated patients was decreased from 148 patients per 100,000 of the Spanish population in 2019 to 118 patients per 100,000 in 2020, possibly due to lack of diagnosis. This is concerning, as COVID-19 lockdown and SARS-CoV-2 infection were shown to produce and aggravate cognitive symptoms, presumably leading to a higher incidence of dementia [42, 43]. The impact of COVID-19 on new AD diagnoses was more pronounced in women than in men, possibly because the greater age and fragility of the former prevented diagnosis and AD-specific treatment. In a recent investigation, older age and female sex were associated with decreased dementia diagnosis during the 2020 pandemic year [44]. Of note, we did not observe a significant decrease in the mean number of treated patients (3954 patients in 2019 to 3910 in 2020, 1.1% decrease), which suggests that the existing treatments were maintained.

The present study had several limitations. Firstly, AD prevalence was estimated based on medication prescription, rather than physician’s etiological diagnosis. Hence, the prevalence might have been underestimated due to not diagnosed or treated patients, but also overestimated, due to treatment of non-AD dementia patients [45]. Nonetheless, our prevalence results were congruent with previous face-to-face investigations [25] and should be valid regarding the evolution of AD diagnosis and treatment during the study period, as well as the COVID-19 impact. Secondly, the lack of etiological diagnosis and analysis adjustment for key control variables (i.e., age and comorbidities) prevented a more complete understanding of patient management. Thirdly, since data collection was finished at the end of the first pandemic year, the consequences of COVID-19 in dementia diagnosis and treatment could not be further described. A recent study demonstrated a lack of global rebound of new dementia cases during 2021, perhaps due to the dramatic increase in mortality suffered by very aged people, while dementia cases ascertained in hospitals and among individuals with multiple comorbidities were higher than expected [46]. Clearly, continued population-based monitoring is needed to fully understand the long-term effects of post-acute SARS-CoV-2 in people with Alzheimer and other dementias.

### Supplementary Information

**Additional file 1: Table S1.** Age-standardized prevalence of AD in total Spanish population. AD: Alzheimer’s disease. **Table S2.** Age-standardized prevalence of AD in female Spanish population. AD: Alzheimer’s disease. **Table S3.** Age-standardized prevalence of AD in male Spanish population. AD: Alzheimer’s disease. **Figure S1.** Comparison between EMR and national population. ^*^EMR population is represented as bars and national population (year 2020) as overlay shadow. EMR: Electronic Medical Records; INE: National Institute of Statistics. **Figure S2.** Groups and frequency of comorbidities in AD-prevalent patients (2013-2020 period, *n*=5,001 prevalent patients).

## Data Availability

The data that support the findings of this study are available from IQVIA, but restrictions apply to the availability of these data, which were used under license for the current study, and so are not publicly available. Data are however available from the authors upon reasonable request and with permission of IQVIA.
