# Supplementary material for: Prevalence of treated patients with Alzheimer’s disease: current trends and COVID-19 impact
Source: Alzheimers Res Ther. 2023 Aug 3;15:130. doi: 10.1186/s13195-023-01271-0 (PMC10401753; doi:10.1186/s13195-023-01271-0)
Supplement: Supplementary file 1 — Additional file 1: Table S1. Age-standardized prevalence of AD in total Spanish population. AD: Alzheimer’s disease. Table S2. Age-standardized prevalence of AD in female Spanish population. AD: Alzheimer’s disease. Table S3. Age-standardized prevalence of AD in male Spanish population. AD: Alzheimer’s disease. Figure S1. Comparison between EMR and national population. *EMR population is represented as bars and national population (year 2020) as overlay shadow. EMR: Electronic Medical Records; INE: National Institute of Statistics. Figure S2. Groups and frequency of comorbidities in AD-prevalent patients (2013-2020 period, n=5,001 prevalent patients). [file 13195_2023_1271_MOESM1_ESM.docx]

SUPPLEMENTARY MATERIALS

Table S1. Age-standardized prevalence of AD in total Spanish population. AD: Alzheimer’s disease.

| **Age group** | **European Standard Population (ESP)** | **Total AD cases (2020)** | **Total population in Spain (2020)** | **Age-standardized AD cases (x10^5^)** |
| --- | --- | --- | --- | --- |
| 0-39 | 47000 | 49 | 20394697 | 11292,15 |
| 40-49 | 14000 | 205 | 7891737 | 36367,15 |
| 50-59 | 13500 | 2616 | 7033306 | 502125,17 |
| 60-69 | 11500 | 15708 | 5336986 | 3384719,39 |
| 70-79 | 9000 | 97931 | 3960045 | 22256792,54 |
| 80-89 | 4000 | 192257 | 2269487 | 33885543,30 |
| ≥90 | 1000 | 36039 | 564537 | 6383815,41 |

Table S2. Age-standardized prevalence of AD in female Spanish population. AD: Alzheimer’s disease

| **Age group** | **European Standard Population (ESP)** | **Female AD cases (2020)** | **Female population in Spain (2020)** | **Age-standardized AD cases (x10^5^)** |
| --- | --- | --- | --- | --- |
| 0-39 | 47000 | 49 | 10010285 | 23006,34 |
| 40-49 | 14000 | 54 | 3898775 | 19390,71 |
| 50-59 | 13500 | 1567 | 3552733 | 595443,00 |
| 60-69 | 11500 | 8234 | 2773107 | 3414617,61 |
| 70-79 | 9000 | 58091 | 2165744 | 24140387,78 |
| 80-89 | 4000 | 126115 | 1394093 | 36185534,25 |
| ≥90 | 1000 | 26929 | 400468 | 6724382,47 |

Table S3. Age-standardized prevalence of AD in male Spanish population. AD: Alzheimer’s disease

| **Age group** | **European Standard Population (ESP)** | **Male AD cases (2020)** | **Male population in Spain (2020)** | **Age-standardized AD cases (x10^5^)** |
| --- | --- | --- | --- | --- |
| 0-39 | 47000 | 0 | 10384412 | 0 |
| 40-49 | 14000 | 151 | 3992962 | 52943,15348 |
| 50-59 | 13500 | 1049 | 3480573 | 406872,6615 |
| 60-69 | 11500 | 7474 | 2563879 | 3352381,294 |
| 70-79 | 9000 | 39840 | 1794301 | 19983269,25 |
| 80-89 | 4000 | 66142 | 875394 | 30222733,99 |
| ≥90 | 1000 | 9110 | 164069 | 5552541,918 |


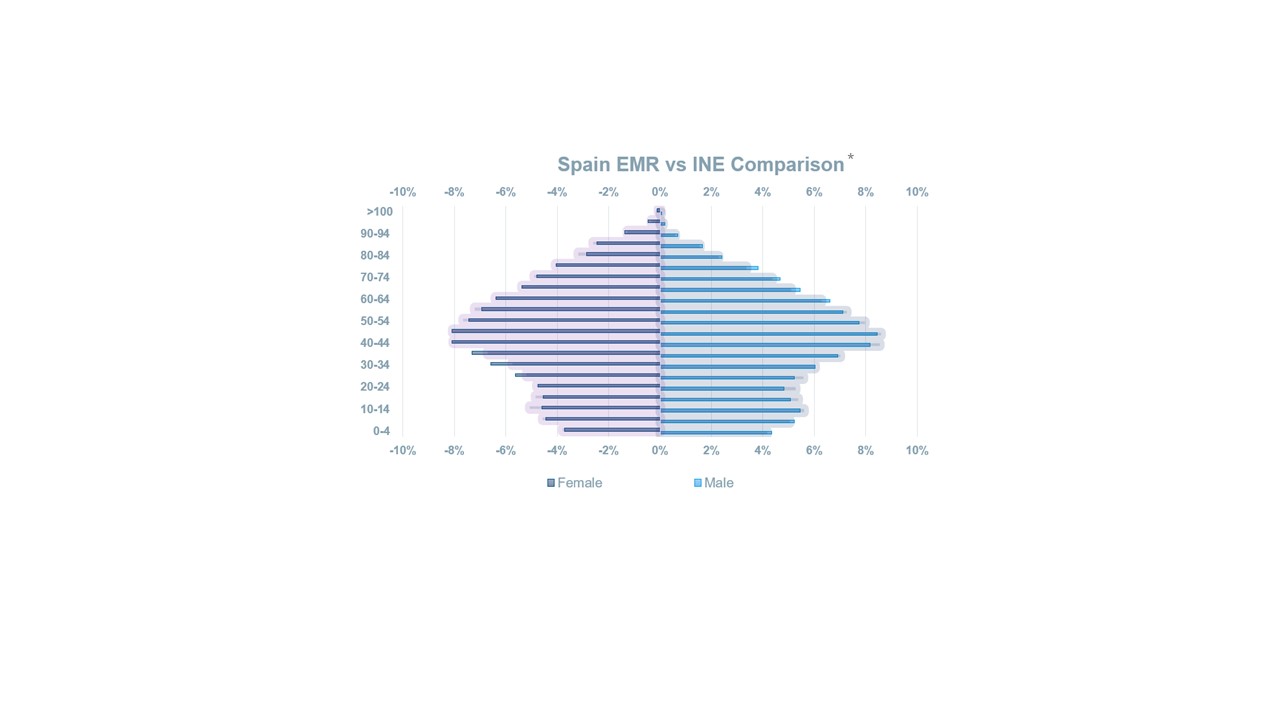


Figure S1. Comparison between EMR and national population. ^*^EMR population is represented as bars and national population (year 2020) as overlay shadow. EMR: Electronic Medical Records; INE: National Institute of Statistics.


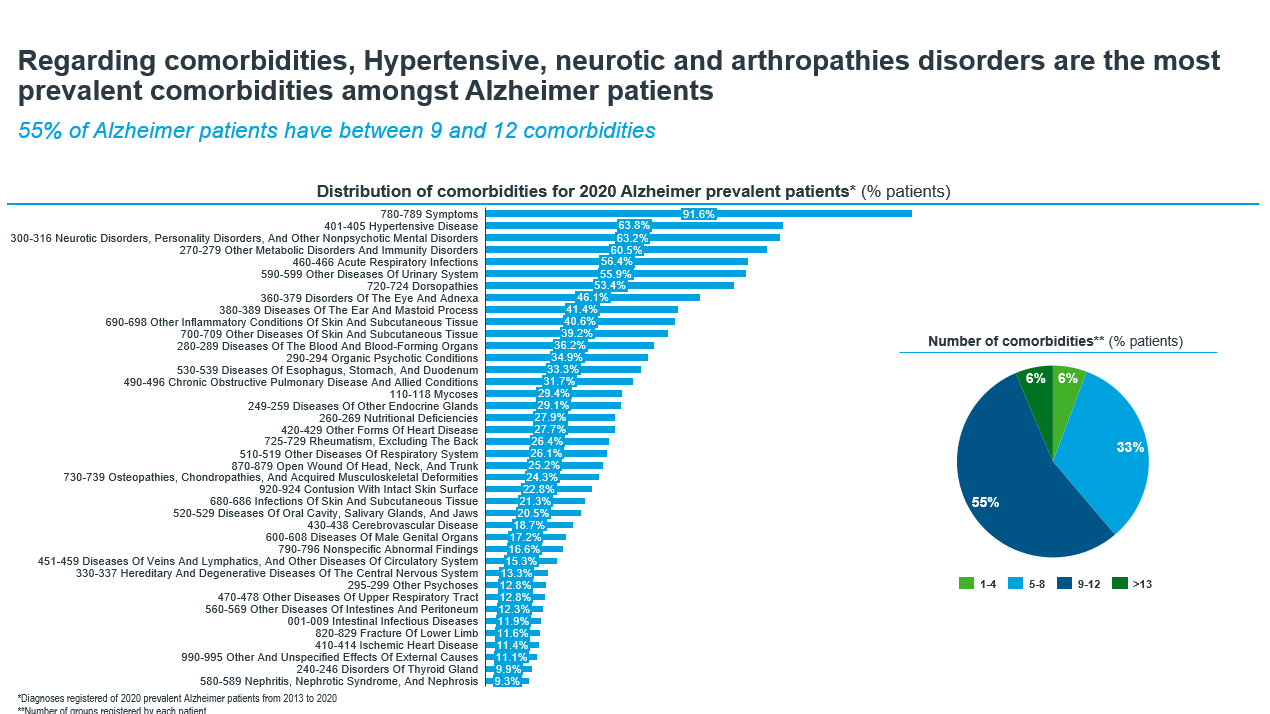


Figure S2. Groups and frequency of comorbidities in AD-prevalent patients (2013-2020 period, n=5,001 prevalent patients)
